# Glypican-3 Expression in Patients with Oral Squamous Cell Carcinoma

**DOI:** 10.30476/DENTJODS.2019.84541.1089

**Published:** 2020-06

**Authors:** Azadeh Andisheh-Tadbir, Amir Saeed Goharian, Mohammad Ali Ranjbar

**Affiliations:** 1 Oral and Dental Disease Research Center, Dept. of Oral and Maxillofacial Pathology, School of Dentistry, Shiraz University of Medical Sciences, Shiraz, Iran.; 2 Undergraduate Student, School of Dentistry, Shiraz University of Medical Sciences, Shiraz, Iran.; 3 Dept. of Oral and Maxillofacial Pathology, School of Dentistry, Shiraz University of Medical Sciences, Shiraz, Iran.

**Keywords:** Glypican-3, Immunohistochemistry, Squamous cell carcinoma

## Abstract

**Statement of the Problem:**

Oral squamous cell carcinoma (OSCC) is a malignant neoplasm that affects the structures or tissues of mouth. Early diagnosis of these tumors is important to improve the outcome of treatment. Therefore, using pathological techniques based on molecular markers may be useful for optimal diagnosis and treatment. Glypican-3 (GPC3) is involved in regulation of cell proliferation and morphogenesis and is abundant during embryogenesis and organogenesis but is limited in most of adult tissues. GPC3 overexpression has a role in carcinogenesis.

**Purpose:**

The aim of the present study was to investigate GPC3 expression in the non-neoplastic oral epithelium and oral squamous cell carcinoma.

**Materials and Method:**

In this cross-sectional study, 45 patients with OSCC (30 males and 15 females) with a mean age of 52.3 selected from Oral Pathology Department of Shiraz Dental School were enrolled. The control group was consisted of 15 cases of normal oral epithelium. Glypican-3 expression was assessed by using immunohistochemical methods.

**Results:**

Non neoplastic tissues were GPC3 negative. Frequency of GPC3 positivity in tumoral tissues was recorded as 73.3% (33 cases) which was significantly higher than non-neoplastic tissues (*p*< 0.001).The clinicopathologic features of GPC3 expression demonstrated no association with clinicopathologic parameters except tumor size.

**Conclusion:**

GPC3 was over expressed at protein level in oral squamous cell carcinoma, but its potential use for diagnostic and therapeutic purposes requires further investigation.

## Introduction

Squamous cell carcinoma is one of the most commonly malignant tumors in the head and neck regions with a locally invasive behavior and highest mortality rate due to malignancies [ 1 ]. These cancers often revealed a clinical diagnostic challenge, especially in its early stages of growth. Attempts are being made for early diagnosis and prevention of this deadly cancer to improve its outcomes. Despite the current improvements in the treatment of this disease, OSCC is a disorder with a high mortality rate and its 5-year survival rate is still poor [ 2 ], therefore, special attention has recently been focused on the use of molecular biomarkers as reliable diagnostic tools of tumors [ 3 ].

GPC3, a cell surface protein, is a member of glypican family and belongs to a group of heparin sulphate proteoglycan bound to the cell membrane through a glycosyl-phosphatidylinositol anchor [ 4 - 5 ]. In mammals, glypican family consists of six membranous, GPC1 to GPC6 [ 6 ]. All glypicans have a common structure that includes a glycosylphosphatidyl inositol anchor to the cell membrane, an N-terminal globular cysteine-rich domain and a C-terminal heparin sulfate glycosaminoglycan attachment site [ 4 , 7 ]. 

In humans, the gene which codes GPC3 is localized to the X chromosome (Xq26) and its product can interact with different molecules such as fibroblast growth factor2, tissue factor pathway inhibitor, Wnt5a and bone morphogenic protein 7 [ 8 - 10 ]. GPC3 regulates the cell proliferation and morphogenesis and is abundant and during embryogenesis and organogenesis [ 11 ], but is limited in most of adult tissues [ 12 - 14 ]. 

GPC3 overexpression has a role in carcinogenesis, particularly in hepatocellular carcinoma [ 9 , 15 - 22 ] but recently it was apparent that GPC3 expression is involved in different extra-hepatic malignant tumors including malignant melanoma [ 23 ], pulmonary squamous cell carcinoma [ 13 ], Merkel cell carcinoma [ 24 ], and chromophobe renal cell carcinoma [ 25 ]. 

The aim of the present study was to investigate GPC3 expression in the non-neoplastic oral epithelium and OSCC by using immunohistochemical methods and to explain the relation of it expression and clinicopathologic features. 

## Materials and Method

In this cross-sectional study, a total of 45 OSCC paraffin blocks were selected from Oral Pathology Department of Shiraz Dental School. The control group was consisted of 15 cases of normal oral epithelium near the lesions. 

Firstly, H & E slides of available blocks were evaluated to confirm the diagnosis; adequate cellular tissues were selected for immunohistochemical staining (IHC). IHC staining was performed by using EnVsion system (DAKO, Carpentaria, CA, USA). All the samples have been fixed in 10% buffered formalin and embedded in paraffin. Sections with 4μ thickness were prepared, deparaffinized in xylene, rehydrated in graded alcohol and washed with distilled water. Antigen retrieval was performed using DakoCytomation target retrieval solution, with pH = 9, for 20 minutes. Internal peroxidase activity was inhibited by 3% H_2_O_2_. Tissue sections were incubated for 30 minutes with the anti-glypican-3 antibody (Abcam, ab66596) at a 1/100 dilution.

Normal samples were stained with the same amount of antibody used for staining tumoral tissues. Gastric epithelium was used as positive control. Negative control was obtained by omission of primary antibody. Brown membranous and cytoplasmic staining for glypican-3 was considered as positive. The slides were assessed under a light microscope (Olympus CX31; Tokyo, Japan) at 400× magnification. The percentage of the positive tumor cells out of 1000 tumoral cells in 5 different fields at high magnification (x400) was calculated and the mean percentage per slide was evaluated. A tumor was considered positive for GPC3 if more than 10% of the neoplastic cells have shown strong cytoplasmic and/or membranous reactivity [ 26 ].

Chi-square test was used to compare the results between the two groups and the relation with clinicopathologic features. 

## Results

**Figure 1:**
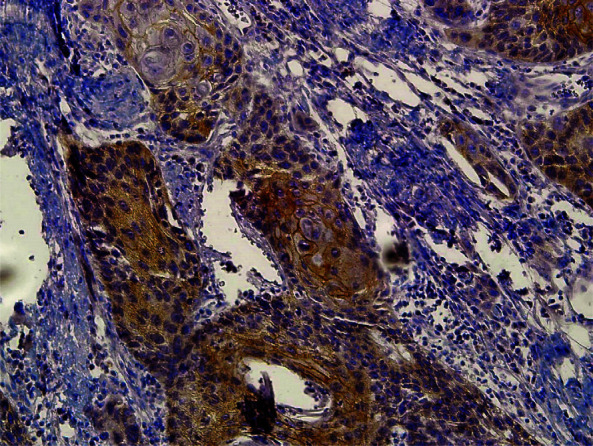
Cytoplasmic and membranous expression of GPC3 in oral squamous cell carcinoma (200×)

**Figure 2:**
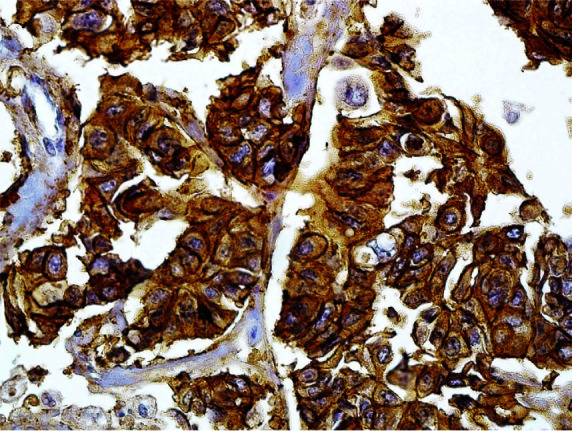
Cytoplasmic and membranous expression of GPC3 in oral squamous cell carcinoma (400×)

**Figure 3:**
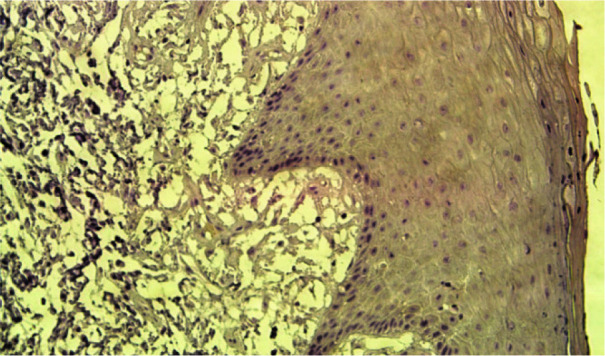
Negative expression of GPC3 in normal oral epithelium (200×)

Gender of patients with OSCC included 15 females (33.3%) and 30 males (66.7%) with a mean age of 52.3± 10.7 years. Control group consisted of 8 females (53.3%) and 7 males (46.6%) with a mean age of 53.6± 10.1 years. In our study, both membranous and cytoplasmic expression of GPC3 was observed in tumoral tissues (Figure 1 and 2). Non neoplastic tissues were GP-C3 negative (Figure 3). GPC3 positivity in tumoral tissues was recorded as 73.3% (33 cases) which was significantly higher than non-neoplastic tissues (*p*< 0.001). Statistically, the expression of GPC3 was significantly higher in samples with larger tumor size (*p*= 0.03). In contrast, there was no significant association between GPC3 expression and other clinico-pathological variables such as clinical staging, grading and lymph node metastasis (*p*> 0.05) (Table 1).

**Table1:** Comparative GPC3 positivity in patients with different clinicopathologic characteristics

Variable	N (%)	Glypican-3	Glypican-3	** Value
		Negative N (%)	Positive N (%)	
T Status				
T1+T2	35(77.7)	12(34.3)	23(65.7)	0.03
T3+T4	10(22.3)	0(0)	10(100)	
N Status				
N0	17(37.7)	3(17.6)	14(82.4)	0.2
N1	28(62.3)	9(32.1)	19(67.9)	
Stage				
I+II	25(55.5)	9(36)	16(64)	0.1
III+IV	20(44.5)	3(15)	17(85)	
Grade				
I	27(60)	9(33.3)	18(66.7)	0.2
II+ III	18(40)	3(16.7)	15(83.3)	

## Discussion

GPC3 is an oncofetal gene that encodes a heparin sulfate proteoglycan that is anchored to the plasma membrane [ 4 ]. GPC3 has a function as a regulator of cell proliferation and morphogenesis [ 11 ] and has a key role in regulating the balance between cell death and cell growth, and is involved in cell apoptosis and cell signal transduction [ 19 , 27 ]. GPC3 is widely expressed in fetal and placental cells during embryonic developmental and organogenesis [ 27 - 32 ] but it disappears in most adult tissue under normal condition [ 15 ]. GPC3can be an important cause of tumorgenesis [ 4 - 5 ], and recent studies demonstrated GPC3 to be a multifunctional proteoglycan molecule with different roles in various diseases [ 33 ]. GPC3 expression is down regulated in lung adenocarcinoma and clear cell renal carcinoma [ 34 - 35 ] but overexpression occurs in hepatocellular carcinoma, ovarian clear cell carcinoma, melanoma, and neuroblastoma [ 14 , 24 , 36 - 37 ]. 

In the present study, GPC3 overexpression was seen in OSCC in comparison to normal tissue which demonstrates the role of GPC3 in the carcinogenesis of OSCCs. Our result was in accordance with previous studies, which demonstrates positive staining of GPC3 in SCC of various sites including lung, cervical, dermal, esophagus, larynx, and anal canal [ 13 , 38 - 39 ]. 

Aviel-Ronen *et al*. [ 38 ] evaluated the expression of GPC3 in lung adenocarcinoma and showed that none of the normal lung tissues stained positively for GPC3. Similar to our findings, there was no association between GPC3 expression and clinicopathological features such as age, gender, stage and outcome. In agreement with our results, another study demonstrated the expression of GPC3 in clear cell carcinoma of ovary and reported no correlation between the expression of GPC3 and clinicopathological factors, like age, gender, stage, and mortality rate, except tumor size [ 14 ]. 

Gonzalez *et al*. [ 33 ] suggested that GPC-3 can inhibit cell proliferation and has a role as a tumor-suppressor gene. Currently, and the role of GPC3 in tumorigenesis and its biological functions is poorly understood and many possible mechanisms regulated by GPC3 during tumorigenesis and tumor progression should be suggested [ 40 ]. The role of GPC3 in cell proliferation and survival may be due to its interaction with insulin-like growth factor-2 [ 41 ]. 

Song *et al*. [ 10 ] showed that Wnt signaling pathway was changed in knockout mice. GPC3 can promote tumor growth by stimulation of canonical Wnt signaling via making a complex with Wnt molecules [ 8 ]. GPC3 can also regulate developmental growth via interaction with the hedgehog signaling pathway [ 42 ]. It can also regulate Bax and Bcl2protein, which are involved in the apoptosis signaling pathways [ 43 ]. Stronger GPC3 expression in the hepatocellular carcinoma would increase epithelial-mesenchymal transition of tumoral cells via interaction with the extracellular signal-regulated kinase (ERK) signaling pathway [ 44 ]. 

Recent research has demonstrated that GPC3 is involved in the proliferation, differentiation, and adhesion of tumor cells, so it has a significant role in tumor growth and metastasis. GPC3 overexpression has been associated with increased tumor growth and metastatic ability [ 45 - 46 ]. There is another study which demonstrates the possible role of GPC3 in malignant transformation of salivary gland tumors. Higher expression of GPC3 in malignant salivary gland tumors in comparison with benign salivary gland tumors showed in this investigation. It may reveal the role of GPC3 in development and invasion of cancers [ 47 ]. 

In the current study, the expression of GPC3 was correlated only with tumor size. GPC3 was currently evaluated as a potential target for tumor specific therapy and immunotherapy [ 48 - 49 ]. Many studies demonstrated that GPC3 peptide vaccine triggers immune response in the patients with advanced hepatocellular carcinoma and the level of immune response was associated with overall survival [ 50 - 51 ]. 

GPC3 expression observed in OSCCs proposed that GPC3 derived vaccine might have an immunotherapeutic application in these tumors. Most of the GPC3 expression pattern in OSCC cases was mixed pattern (cytoplasmic and membranous). The functional difference between two different patterns GPC3 expression (cytoplasmic and membranous) is unrevealed, therefore, more studies are recommended to determine the significance of different localization of GPC3. GPC3 can be secreted from tumoral cells and be detected in the serum of the patients [ 52 - 53 ]. GPC3 was shown to be a valid tumor marker of hepatocellular carcinoma, which can be used for early detection of patients with hepatocellular carcinoma by blood screening [ 6 , 52 - 53 ].Hence, further investigations are needed to evaluate the association between GPC3 expression and its serum level.

## Conclusion

GPC3 was overexpressed at protein level in OSCC; there was no association between GPC3 expression and clinicopathologic parameters except tumor size. Therefore, its potential use for diagnostic and therapeutic purposes requires further investigation. 

## References

[1] Monteiro LS, Diniz-Freitas M, Warnakulasuriya S, Garcia-Caballero T, Forteza J, Fraga M ( 2018). An immunohistochemical score to predict the outcome for oral squamous cell carcinoma. J Oral Pathol Med.

[2] Epstein JB, Wan LS, Gorsky M, Zhang L ( 2003). Oral lichen planus: progress in understanding its malignant potential and the implications for clinical management. Oral Surg Oral Med Oral Pathol Oral Radiol Endod.

[3] Mithani SK, Mydlarz WK, Grumbine FL, Smith IM, Califano JA ( 2007). Molecular genetics of premalignant oral lesions. Oral Dis.

[4] Filmus J, Selleck SB ( 2001). Glypicans: proteoglycans with a surprise. J Clin Invest.

[5] Wang F, Jing X, Wang T, Li G, Li T, Zhang Q ( 2012). Differential diagnostic value of GPC3-CD34 combined staining in small liver nodules with diameter less than 3 cm. Am J Clin Pathol.

[6] Hippo Y, Watanabe K, Watanabe A, Midorikawa Y, Yamamoto S, Ihara S ( 2004). Identification of soluble NH2-terminal fragment of glypican-3 as a serological marker for early-stage hepatocellular carcinoma. Cancer Res.

[7] Sasisekharan R, Shriver Z, Venkataraman G, Narayanasami U ( 2002). Roles of heparan-sulphate glycosaminoglycans in cancer. Nat Rev Cancer.

[8] Capurro MI, Xiang YY, Lobe C, Filmus J ( 2005). Glypican-3 promotes the growth of hepatocellular carcinoma by stimulating canonical Wnt signaling. Cancer Res.

[9] Midorikawa Y, Ishikawa S, Iwanari H, Imamura T, Sakamoto H, Miyazono K ( 2003). Glypican-3, overexpressed in hepatocellular carcinoma, modulates FGF2 and BMP-7 signaling. Int J Cancer.

[10] Song HH, Shi W, Xiang YY, Filmus J ( 2005). The loss of glypican-3 induces alterations in Wnt signaling. J Biol Chem.

[11] Grozdanov PN, Yovchev MI, Dabeva MD ( 2006). The oncofetal protein glypican-3 is a novel marker of hepatic progenitor/oval cells. Lab Invest.

[12] Pellegrini M, Pilia G, Pantano S, Lucchini F, Uda M, Fumi M ( 1998). Gpc3 expression correlates with the phenotype of the Simpson-Golabi-Behmel syndrome. Dev Dyn.

[13] Baumhoer D, Tornillo L, Stadlmann S, Roncalli M, Diamantis EK, Terracciano LM ( 2008). Glypican 3 expression in human nonneoplastic, preneoplastic, and neoplastic tissues: a tissue microarray analysis of 4,387 tissue samples. Am J Clin Pathol.

[14] Maeda D, Ota S, Takazawa Y, Aburatani H, Nakagawa S, Yano T ( 2009). Glypican-3 expression in clear cell adenocarcinoma of the ovary. Mod Pathol.

[15] Man XB, Tang L, Zhang BH, Li SJ, Qiu XH, Wu MC ( 2005). Upregulation of Glypican-3 expression in hepatocellular carcinoma but downregulation in cholangiocarcinoma indicates its differential diagnosis value in primary liver cancers. Liver Int.

[16] Filmus J, Capurro M ( 2008). The role of glypican-3 in the regulation of body size and cancer. Cell Cycle.

[17] Kwack MH, Choi BY, Sung YK ( 2006). Cellular changes resulting from forced expression of glypican-3 in hepatocellular carcinoma cells. Mol Cells.

[18] Nakatsura T, Nishimura Y ( 2005). Usefulness of the novel oncofetal antigen glypican-3 for diagnosis of hepatocellular carcinoma and melanoma. BioDrugs.

[19] Lü ZL, Luo DZ, Wen JM ( 2005). Expression and significance of tumor-related genes in HCC. World J Gastroenterol.

[20] Li BD, Zhao QC, Zhu YT, Zhang FQ, Dou KF ( 2006). Significance of glypican-3 mRNA expression in hepatocellular carcinoma tissues and peripheral blood cells. Zhonghua Wai Ke Za Zhi.

[21] Zhu ZW, Friess H, Wang L, Abou-Shady M, Zimmermann A, Lander AD ( 2001). Enhanced glypican-3 expression differentiates the majority of hepatocellular carcinomas from benign hepatic disorders. Gut.

[22] Akutsu N, Yamamoto H, Sasaki S, Taniguchi H, Arimura Y, Imai K ( 2010). Association of glypican-3 expression with growth signaling molecules in hepatocellular carcinoma. World J Gastroenterol.

[23] Nakatsura T, Kageshita T, Ito S, Wakamatsu K, Monji M, Ikuta Y ( 2004). Identification of glypican-3 as a novel tumor marker for melanoma. Clin Cancer Res.

[24] He H, Fang W, Liu X, Weiss LM, Chu PG ( 2009). Frequent expression of glypican-3 in Merkel cell carcinoma: an immunohistochemical study of 55 cases. Appl Immunohistochem Mol Morphol.

[25] Okoń K (2008). Glypican-3 is expressed in chromophobe renal cell carcinoma. Pol J Pathol.

[26] Mounajjed T, Zhang L, Wu TT ( 2013 Apr). Glypican-3 expression in gastrointestinal and pancreatic epithelial neoplasms. Hum Pathol.

[27] Umezu T, Shibata K, Shimaoka M, Kajiyama H, Yamamoto E, Ino K ( 2010). Gene silencing of glypican-3 in clear cell carcinoma of the ovary renders it more sensitive to the apoptotic agent paclitaxel in vitro and in vivo. Cancer Sci.

[28] Khan S, Blackburn M, Mao DL, Huber R, Schlessinger D, Fant M ( 2001). Glypican-3 (GPC3) expression in human placenta: localization to the differentiated syncytiotrophoblast. Histol Histopathol.

[29] Liu B, Paranjpe S, Bowen WC, Bell AW, Luo JH, Yu YP ( 2009). Investigation of the role of glypican 3 in liver regeneration and hepatocyte proliferation. Am J Pathol.

[30] Pantanowitz L, Otis CN ( 2008). Glypican-3 immunohistochemistry in the ovary. Histopathology.

[31] Ushiku T, Uozaki H, Shinozaki A, Ota S, Matsuzaka K, Nomura S ( 2009). Glypican 3-expressing gastric carcinoma: distinct subgroup unifying hepatoid, clear-cell, and alpha-fetoprotein-producing gastric carcinomas. Cancer Sci.

[32] Iglesias BV, Centeno G, Pascuccelli H, Ward F, Peters MG, Filmus J ( 2008). Expression pattern of glypican-3 (GPC3) during human embryonic and fetal development. Histol Histopathol.

[33] Gonzalez AD, Kaya M, Shi W, Song H, Testa JR, Penn LZ ( 1998). OCI-5/GPC3, a glypican encoded by a gene that is mutated in the Simpson-Golabi-Behmel overgrowth syndrome, induces apoptosis in a cell line-specific manner. J Cell Biol.

[34] Kim H, Xu GL, Borczuk AC, Busch S, Filmus J, Capurro M ( 2003). The heparan sulfate proteoglycan GPC3 is a potential lung tumor suppressor. Am J Respir Cell Mol Biol.

[35] Valsechi MC, Oliveira AB, Conceição AL, Stuqui B, Candido NM, Provazzi PJ ( 2014). GPC3 reduces cell proliferation in renal carcinoma cell lines. BMC Cancer.

[36] Li YS, Milner PG, Chauhan AK, Watson MA, Hoffman RM, Kodner CM ( 1990). Cloning and expression of a developmentally regulated protein that induces mitogenic and neurite outgrowth activity. Science.

[37] Stadlmann S, Gueth U, Baumhoer D, Moch H, Terracciano L, Singer G ( 2007). Glypican-3 expression in primary and recurrent ovarian carcinomas. Int J Gynecol Pathol.

[38] Aviel-Ronen S, Lau SK, Pintilie M, Lau D, Liu N, Tsao MS, Jothy S ( 2008). Glypican-3 is overexpressed in lung squamous cell carcinoma, but not in adenocarcinoma. Mod Pathol.

[39] Nemolato S, Fanni D, Naccarato AG, Ravarino A, Bevilacqua G, Faa G ( 2008). Lymphoepithelioma-like hepatocellular carcinoma: A case report and a review of the literature. World J Gastroenterol.

[40] Zou S, Cao N, Cheng D, Zheng R, Wang J, Zhu K ( 2012). Enhanced apoptosis of ovarian cancer cells via nanocarrier- mediated codelivery of siRNA and doxorubicin. Int J Nanomedicine.

[41] Ho M, Kim H ( 2011). Glypican-3: a new target for cancer immunotherapy. Eur J Cancer.

[42] Capurro MI, Xu P, Shi W, Li F, Jia A, Filmus J ( 2008). zlypican-3 inhibits Hedgehog signaling during development by competing with patched for Hedgehog binding. Dev Cell.

[43] Srivastava K, Srivastava A, Mittal B ( 2013). Potential biomarkers in gallbladder cancer: present status and future directions. Biomarkers.

[44] Qi XH, Wu D, Cui HX, Ma N, Su J, Wang YT ( 2014). Silencing of the glypican-3 gene affects the biological behavior of human hepatocellular carcinoma cells. Mol Med Rep.

[45] Tanaka SS, Kojima Y, Yamaguchi YL, Nishinakamura R, Tam PP ( 2011). Impact of WNT signaling on tissue lineage differentiation in the early mouse embryo. Dev Growth Differ.

[46] Gao W, Ho M ( 2011). The role of glypican-3 in regulating Wnt in hepatocellular carcinomas. Cancer Rep.

[47] Andisheh-Tadbir A, Ashraf MJ, Gudarzi A, Zare R ( 2019). Evaluation of Glypican-3 expression in benign and malignant salivary gland tumors. Oral Biol Craniofac Res.

[48] De Cat B, Muyldermans SY, Coomans C, Degeest G, Vanderschueren B, Creemers J ( 2003). Processing by proprotein convertases is required for glypican-3 modulation of cell survival, Wnt signaling, and gastrulation movements. J Cell Biol.

[49] Filmus J, Capurro M ( 2013). Glypican-3: a marker and a therapeutic target in hepatocellular carcinoma. FEBS J.

[50] Komori H, Nakatsura T, Senju S, Yoshitake Y, Motomura Y, Ikuta Y ( 2006). Identification of HLA-A2- or HLA-A24-restricted CTL epitopes possibly useful for glypican-3-specific immunotherapy of hepatocellular carcinoma. Clin Cancer Res.

[51] Sawada Y, Yoshikawa T, Nobuoka D, Shirakawa H, Kuronuma T, Motomura Y ( 2012). Phase I trial of a glypican- 3-derived peptide vaccine for advanced hepatocellular carcinoma: immunologic evidence and potential for improving overall survival. Clin Cancer Res.

[52] Capurro M, Wanless IR, Sherman M, Deboer G, Shi W, Miyoshi E, Filmus J ( 2003). Glypican-3: a novel serum and histochemical marker for hepatocellular carcinoma. Gastro enterology.

[53] Nakatsura T, Yoshitake Y, Senju S, Monji M, Komori H, Motomura Y ( 2003). Glypican-3, overexpressed specifically in human hepatocellular carcinoma, is a novel tumor marker. Biochem Biophys Res Commun.

